# Tablet vs. station-based laptop ultrasound devices increases internal medicine resident point-of-care ultrasound performance: a prospective cohort study

**DOI:** 10.1186/s13089-020-00165-8

**Published:** 2020-04-16

**Authors:** Matt Glogoza, Jonathan Urbach, Terry K. Rosborough, Susan Olet, Catherine A. St. Hill, Claire S. Smith, David M. Tierney

**Affiliations:** 1 Department of Graduate Medical Education #11135, Abbott Northwestern Hospital, 800 E. 28th Street, Minneapolis, MN 55407 USA; 2 Clinical Research Informatics and Analytics, Allina Health, Minneapolis, MN USA; 3 Department of Care Delivery Research, Allina Health, Minneapolis, MN USA

**Keywords:** Point-of-care ultrasound, Internal medicine, Medical education

## Abstract

**Background:**

Point-of-care ultrasound (POCUS) is becoming an important part of internal medicine (IM) residency training. Achieving competency requires performing a large volume of clinical exams which can be difficult within the constraints of residency. Often-cited barriers include insufficient resident time and the interruption of daily workflow. Despite availability of hospital station-based laptop ultrasound machines, we hypothesized that the addition of ward team-based tablet ultrasound devices would lower barriers and increase clinical POCUS volume within an IM residency POCUS curriculum at a 670-bed, quaternary care, teaching hospital. IM resident POCUS volumes and characteristics during an 18-mo. baseline (station-based laptop devices only) period were compared to matched months during the intervention (station-based + tablet).

**Results:**

Total patients examined with POCUS by 6 inpatient resident teams during the 18-mo. baseline and intervention periods were 1386 and 1853, respectively. Patients examined per month increased during the intervention by 34% (77 vs. 103, *p* = 0.002). The number of areas (e.g., abdominal, cardiac) and items (e.g., bladder, pericardial effusion) examined per month increased by 27% (*p* = 0.021) and 23% (*p* = 0.073), respectively.

**Conclusions:**

A combined program infrastructure of station-based laptop and “in-the-pocket” tablet ultrasound devices lowered common POCUS barriers of inadequate time and workflow disruption for IM residents and resulted in a meaningful increase of exams within a longitudinal residency-based training program where station-based laptop devices already existed.

## Background

Point-of-care ultrasound (POCUS) significantly improves an internist’s diagnostic ability at the bedside and is quickly becoming part of internal medicine (IM) residency training [1–3]. Recent position statements from the Society of Hospital Medicine, American College of Physicians, and Alliance for Academic Internal Medicine all endorse POCUS as an important tool for internists [3–6].

A goal of the American College of Physicians is to “define the educational curriculum needed to train residents and internists in the appropriate use of POCUS in internal medicine” [5]. An IM residency-based curriculum should be longitudinal and target POCUS competency which encompasses basic ultrasound knowledge, image acquisition and identification skills, and the ability to clinically integrate findings. A large volume of clinical exams is essential for residents to understand how to clinically integrate POCUS findings [3]. Barriers to performing clinical exams include lack of time and workflow disruption [7–9]. Successful IM residency POCUS curricula must adopt programmatic efficiencies to overcome these barriers—specifically those targeting clinical exam performance amongst residents’ “own” patients.

The Internal Medicine Bedside Ultrasound (IMBUS) Program, a longitudinal, 3-year, IM residency-based POCUS curriculum began at our hospital in 2010. Portable, laptop ultrasound devices were initially stored in a locked closet in the residency office. In September 2013, cart-based laptop ultrasound devices were introduced to each hospital station. This correlated with an increase in ultrasound use amongst the residents. The objective of this study was to evaluate whether, even in the setting of widely available station-based devices, the addition of tablet ultrasound devices to the pockets of resident inpatient ward teams would further increase the volume of clinical POCUS exams being performed by residents.

## Methods

### Setting

This prospective cohort study took place within a 670-bed, quaternary-care, teaching hospital from 2015 to 2019. The hospital’s 30-resident IM residency program has a long-standing in- and outpatient POCUS curriculum (IMBUS) started in 2010. Ward teams comprise a hospitalist attending, one first-year and one second-year resident, and 2 medical students. The IRB approved the study as non-human subject research (Allina Health IRB; Reference 1070078-1).

### IMBUS curriculum and infrastructure

First-year IM residents participate in a 5-day, 40-h (70% hands-on) POCUS course in August. All resident-performed POCUS exams following that introductory course are mentored by a certified faculty member at the bedside until the resident is certified in the given exam area. Once the minimum quantity requirement within an exam area is obtained (Additional file 1: Digital Content S1. IMBUS Certification Criteria), the resident is assessed at the bedside for the other elements of POCUS competency within that specific area (i.e. interpretation and clinical integration). Once quantity of exams and bedside assessment of competency are achieved, the resident is deemed “certified” in that exam area [10]. First-year residents have a required 1-month ultrasound and procedure team rotation, which is a 1:1 intensive POCUS experience with 3 of our core faculty. Second-year residents participate in an advanced outpatient POCUS rotation in our main faculty primary care clinic [11]. Many of our residents select a 1-month POCUS elective during their 2nd or 3rd year of training.

**Fig. 1:**
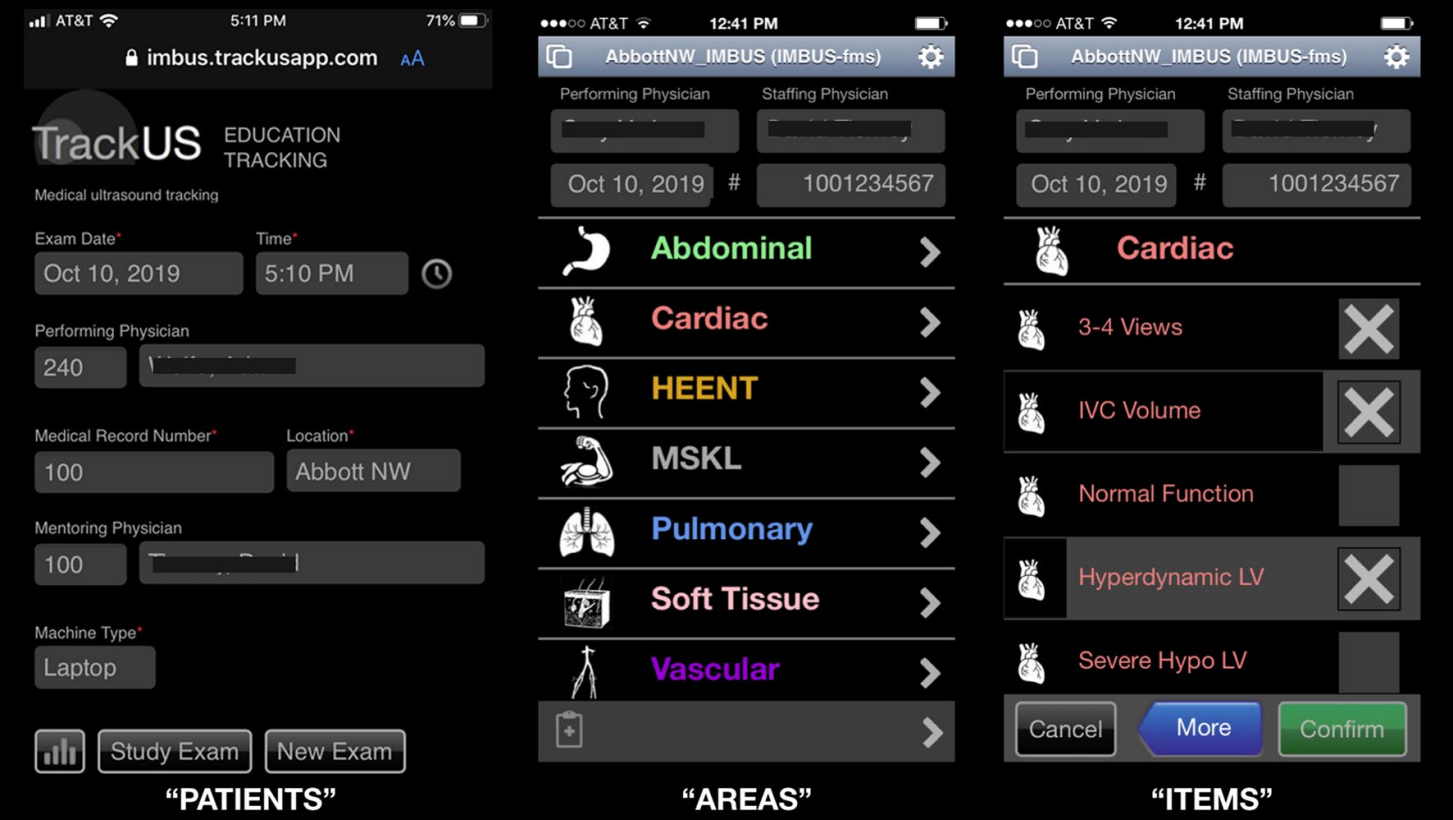
Smartphone-based tracking application used to record “PATIENT” exams, “AREAS” examined on each patient, and “ITEMS” observed in each area

Beginning July 2012, resident and faculty ultrasound exams have been prospectively tracked on a multi-platform, web-based application with an interface on all resident smartphones. The application records type of ultrasound device used, location of exam (hospital or clinics), exam areas and items observed, faculty mentor, etc. (Fig. 1). The data are collected in a database allowing residents real-time access, via the smartphone application, to their current volume of exams in each certification area.

**Fig. 2:**
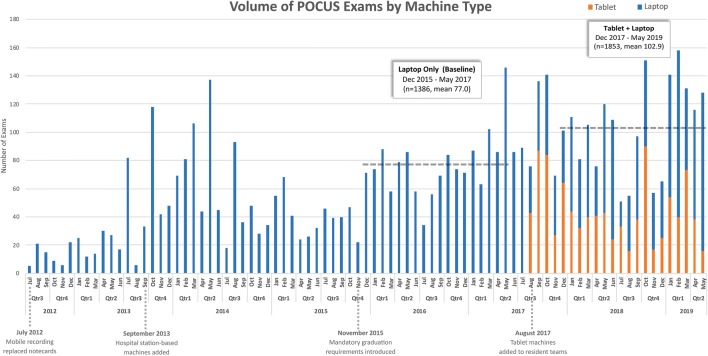
Volume of POCUS exams by month within the IMBUS program, limited to those performed by residents on inpatient rotations. Baseline (station-based laptop ultrasound devices only) period (December 2015–May 2017) and intervention (station-based laptop devices + tablet devices) period (December 2017–May 2019) with horizontal dashed lines representing the mean number of exams for each period (77.0 vs. 102.9). Proportions of exams performed by device type from August 2017 through May 2019 are shown with orange (tablet) vs. blue (laptop) bars. The timeline shown annotates significant historical programmatic changes impacting volume of exams within the IMBUS program

There have been several changes within the IMBUS curriculum since it began in 2010. Many of these have impacted the motivation of and volume of exams being performed by residents and faculty in the program (Fig. 2). Station-based laptop ultrasound devices with dedicated central parking locations on each station were added in September 2013 (SonoSite EDGE devices with P21 [1-5 mHz] phased-array and L25 [13–6 mHz] linear transducers; FUJIFILM SonoSite Inc., Bothell, WA). The devices were available in significant excess of demand, and when a given device was in use, the adjacent station’s device was likely available. A hospital-wide tracking system provided residents real-time, room-specific location data for all devices. A separate fleet of ultrasound devices are used for procedures and educational scanning so as not to tie up the station-based devices. In November 2015, IMBUS certification within the core areas (Additional file 1: Digital Content S1. IMBUS Certification Criteria) became a mandatory graduation requirement (Fig. 2).

In mid-August 2017, an 8-in. tablet able to fit into a white coat pocket (Huawei Technologies Co Ltd., model SHT-W09; Shenzhen, China) and a detachable sector array transducer (Philips Lumify S4-1 [4–1 mHz]; Philips Ultrasound Inc., Bothell, WA) were deployed on each of 6 resident ward teams. The intensive care unit-based resident team also received a linear transducer (Philips Lumify L12-4 [12–4 mHz]; Philips Ultrasound Inc., Bothell, WA). Residents on non-ward inpatient rotations did not have tablet ultrasounds, so continued to use the station-based laptop devices. Between December 2015 and May 2019, there were no other significant changes to the IMBUS curriculum/infrastructure, graduation requirements, resident individual or team characteristics, or inpatient population/census.

### Baseline and intervention period definitions

Baseline data were collected starting after the introduction of graduation requirements in December 2015 through May 2017 (18 mo.), during which only station-based laptop ultrasound devices were available. Due to the known seasonal variation in resident-performed POCUS within the IMBUS program, the intervention period did not include September through November of 2017 (the first 3 months after ward team tablet ultrasound devices were added), but rather included the 18 months with paired baseline data from December 2017 through May 2019 (Fig. 2). All data (including June–October 2017) from December 2015 through May 2019 was used for inflection point analysis.

### Data analysis

The data are presented as counts and proportions for categorical variables and as mean ± standard deviation for continuous variables. A paired t test was used to assess whether there was a difference between total monthly exams performed during baseline and intervention periods. Using the iterative application of geometrical methods, a Unit Invariant Knee (UIK) point—an objective estimator for the “elbow point” was determined using inflection point analysis [12]. The “elbow point” is practically the point where a sharply diminishing or increasing curve starts to become ‘flat enough’. All tests were performed at a 5% level of significance and the statistical analysis was done using SAS version 9.4 and R version 3.6.1 (2019-07-05).

## Results

Total patients that underwent POCUS exams by 6 inpatient resident teams during the 18-mo. baseline and intervention periods were 1386 and 1853, respectively. There is a seasonal variation to the overall volume of POCUS exams performed by residents with predictable nadirs in July and August (prior to new first-year residents being trained, and new second-year residents in the team leader role), and peaks in September (the month after the week-long training course for first-year residents) and late spring (prior to resident advancement and graduation).

When analyzed by paired-month due to seasonal variation, the mean number of patients examined per month increased significantly during the intervention period by 34% (77 vs. 103, *p* = 0.002). The number of areas (e.g., abdominal, cardiac) and items (e.g., bladder, pericardial effusion) examined per month increased by 27% (*p* = 0.021) and 23% (*p* = 0.073), respectively. There was no meaningful difference between baseline and intervention periods for the number of areas or items examined per patient exam, overall, or by ultrasound device type (i.e. laptop vs. tablet).

To further establish that the tablet ultrasound devices were responsible for the significant increase in monthly exam volume between baseline and intervention periods, instead of the observed difference being solely a result of an underlying positive trend present throughout the 39-month study period, two additional analyses were performed. Inflection point analysis [12] demonstrated two separate elbow points across the 39-month period from December 2015 to May 2019. The first occurred at the very large seasonal variation peak at the end of the baseline period in May of 2017 (Fig. 2). When accounting for the impact of May seasonal variation on the inflection analysis, a second observed elbow was identified in September of 2017, corresponding with the introduction of the tablet ultrasound devices. Additionally, monthly volume between the first and second halves of the baseline and subsequently between the first and second halves of the intervention periods were compared and showed no significant increase (or decrease) in volume within either study period (*p* value for differences = 0.275 and 0.335, respectively).

**Fig. 3:**
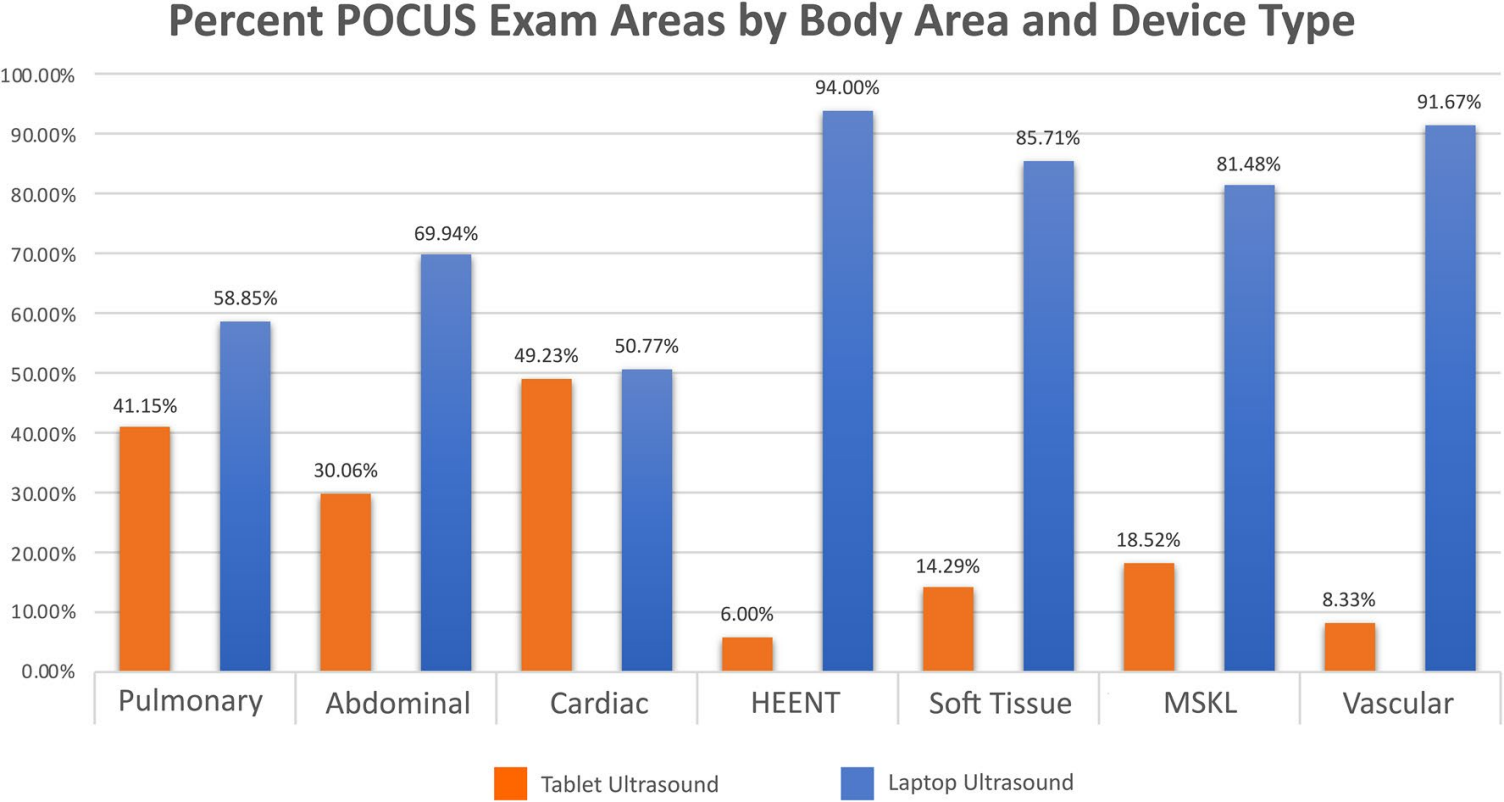
Percent of POCUS exam areas (*n* = 2901) performed grouped by body area and ultrasound device type (tablet vs. laptop) from September 2017 through May 2019. HEENT = head, eyes, ears, nose, throat; MSKL = musculoskeletal

During the timeframe when tablets were present on teams (September 2017–May 2019), 42% (926 vs. 1273) of patient exams were performed using the tablet ultrasound devices. Frequency of tablet vs. laptop utilization for exams areas where a low-frequency (sector) transducer was appropriate was 41.2% vs. 58.9% for pulmonary, 30.1% vs. 69.9% for cardiac, and 49.2% vs. 50.8% for the abdominal area (Fig. 3). The critical care team (the only team with a linear array transducer) had the ability to perform additional tablet-based exams that required a high-frequency transducer. This team accounted for all the tablet-performed exams within the exam areas of HEENT (6%), soft tissue (14%), musculoskeletal (19%), and vascular (8%). All exams in these areas performed by the other 5 ward teams (without linear transducers) were performed using the laptop ultrasound devices.

During the baseline period, an audit of 100 resident-performed exams showed an 87% recording rate. Because it was possible that resident recording of exams may have increased during the intervention period, an analysis theorizing a change to 100% recording during the intervention period was performed demonstrating a maintained 18% increase in exams (*p* = 0.048).

Residents were asked to consider the previous barriers to regularly using laptop ultrasound devices. Frequent responses included: “it just wasn’t on my mind while I was in the room”, “the flow of taking a history and doing a physical exam is disrupted when I leave the room to get a device”, and “I didn’t have time”. When asked why they performed more exams after the introduction of tablet ultrasound devices, resident comments were recorded and categorized into two major themes. The first theme encompassed the idea that a device you carry with you (like your stethoscope) feels more like an intrinsic part of being an internist, compared to getting a common device from the nursing station. A second theme centered around the often-forgotten impact of context shifting on quality and efficiency.

## Discussion

The impact of POCUS on clinical quality and safety, system efficiency, patient and physician satisfaction, and medical education has resulted in a quickly growing rate of adoption within medical schools and IM residency programs [2–4, 13, 14]. However, fully realizing these benefits takes a significant investment in training. The most efficient training setting is within graduate medical education where mentorship, immersion, and condensed repetition can take place. Once programmatic aspects of a residency-based training curriculum are secured, the success of the program becomes dependent on volume of resident-performed clinical exams. That clinical volume is requisite for, but not sufficient for achieving and maintaining competency.

The results of this study show that the addition of “in their pocket” tablet ultrasound devices to 6 IM ward teams significantly increased volume of clinical POCUS exams even in a mature POCUS training environment with abundant station-based ultrasound devices. The IMBUS program trialed several incentive programs from 2012 to 2015 aimed at increasing resident daily POCUS volume. Other than the addition of a mandatory graduation requirement and moving devices from the residency offices to the inpatient stations, no other efforts significantly influenced volume.

The reason for the increased volume seen in this study is unlikely related solely to the few minutes saved by having the device in your pocket versus getting a machine from the nursing station. To fully understand why the addition of tablets impacted volume to the extent seen, it is helpful to consider the previous barriers to regularly using station-based US reported by the residents, but perhaps more insightful to reflect on their ideas behind why they performed more exams after the introduction of the tablet devices. One unanticipated theme, the idea that “a device you carry with you (like your stethoscope) feels more like an intrinsic part of being an internist”, may have provided subtle but significant motivation to master the tool. A second theme, centered around the disruption of context shifting in a resident’s daily workflow where partially completed tasks are frequently put on hold, was likely minimized by not having to leave a patient encounter to retrieve an ultrasound machine. We conjecture that these two impacts, beyond the actual time savings of having an ultrasound in your pocket, likely have a meaningful impact on a resident’s likelihood of performing exams.

There are several reasons why residents did not use the tablet devices for all exams (Fig. 3). First, the tablet devices were only added to the hands of 12 residents (6 two-resident teams) performing POCUS in the hospital. While the vast majority of POCUS exams within the IMBUS program are performed on the inpatient resident ward teams, senior residents on non-ward rotations only had access to laptop devices providing a constant laptop background use across periods. If a resident carrying only a sector transducer (5 of the 6 teams) was going to perform a combined exam necessitating both sector and linear array transducers (e.g., cardiac and vascular exams), they would have likely used the station-based laptop cart. In an educational setting where an additional goal of POCUS is teaching patients, family members, and medical students, the larger screen size of a laptop device is more easily viewed and may be preferentially chosen. Finally, certain cardiac applications within the curriculum utilize spectral and tissue Doppler unavailable on these tablet devices.

Image quality was not directly compared between tablet and laptop devices in this study. However, the fact that only 12 patients (0.6%) initially examined with the tablet ultrasound device had a subsequent exam recorded using a laptop within the same exam area on the same day supports the anectdotal experience that the images obtained using the tablet ultrasound was rarely inadequate for answering the clinical POCUS question being asked. This conclusion is limited as residents may have chosen to initially use a laptop device for patients who they predicted would be inadequately imaged with the tablet or not recorded both exams.

In the current state of ultra-portable ultrasound devices, these study results support a rationale for a mixed fleet of devices in the inpatient setting. The cost and quality of ultra-portable tablet and phone-sized ultrasound devices vary considerably, however a program is often able to purchase 5–20 pocket-size devices for the cost of a cart-based laptop device. Thus, a combination of devices is often the most cost-effective approach, and this study would also support an educational rationale for this approach within an IM inpatient training environment. However, as ultra-portable devices evolve to deliver image quality equivalent to larger devices, features such as spectral Doppler, and the ability to display images on various screen sizes, their current benefits of significantly lower cost and impact on learner volume demonstrated in this study may result in a future rationale for a fleet including only tablet-sized devices in resident coat pockets.

This study has limitations. (1) It took place in a single center’s, mature POCUS training program that has already moved beyond the programmatic barriers often encountered (e.g., faculty mentors, sufficient devices, training time, etc.). In a less mature program, or a program with a different baseline machine workflow, the relative impact of tablet device addition may differ. (2) The number of ultrasound devices available in the hospital increased from 14 to 20 with the addition of the tablet devices during the intervention period. Station-based devices were rarely unavailable during the baseline period, however, we cannot exclude that some of the increase in volume was due to machine availability. (3) Residents were not aware of this study, and even if there was a Hawthorne effect, the sustained > 18 month increase in volume makes an effect unlikely. Further, if the goal is to increase exam volume, these limitations do not necessarily detract from the study conclusions.

## Conclusions

The addition of team-based tablet ultrasound devices in a setting with abundant station-based laptop ultrasound devices, significantly increased inpatient POCUS volume within a 3-year longitudinal, residency-based, IM POCUS curriculum. Especially in the initial training environment where volume of exams is often a limiting factor, the addition of “in your pocket” devices may provide motivation beyond just the time savings of “getting a device” and be an important element in efficiently achieving trainee competency.

## Supplementary information

**Additional file 1: Digital Content S1.** “IMBUS Certification Areas & Items”. Listing of exam items by area that are tracked within the IMBUS program for certification. Minimum number of exams required to be eligible for certification assessment within each exam item is listed. Achievement of minimum quantities is requisite for but not the only aspect required for certification in the item. Core elements noted are required for residency graduation.

## Data Availability

The datasets used and/or analyzed during the current study are available from the corresponding author on reasonable request.

## Abbreviations

POCUS: Point-of-care ultrasound
IMBUS: Internal medicine bedside ultrasound
IM: Internal medicine
