# Supplementary material for: Tablet vs. station-based laptop ultrasound devices increases internal medicine resident point-of-care ultrasound performance: a prospective cohort study
Source: Ultrasound J. 2020 Apr 16;12:18. doi: 10.1186/s13089-020-00165-8 (PMC7163000; doi:10.1186/s13089-020-00165-8)
Supplement: Supplementary file 1 — Additional file 1: Digital Content S1. “IMBUS Certification Areas & Items”. Listing of exam items by area that are tracked within the IMBUS program for certification. Minimum number of exams required to be eligible for certification assessment within each exam item is listed. Achievement of minimum quantities is requisite for but not the only aspect required for certification in the item. Core elements noted are required for residency graduation. [file 13089_2020_165_MOESM1_ESM.pdf]

Supplemental Digital Content 1: "IMBUS Certification Areas & Items"

Listing of exam items by area that are tracked within the IMBUS program for certification. Minimum number of exams required to be eligible for certification assessment within each exam item is listed. Achievement of minimum quantities is requisite for but not the only aspect required for certification in the item. Core elements noted are required for residency graduation.

Exam Area	Exam Item	Certification Cutpoint	Core Element
**ABDOMINAL**			
	Liver Size	8	x
	Hepatomegaly	5	x
	Morrison's	8	x
	Liver Mass	3	
	Liver Steatosis	3	
	Ascites	5	x
	Spleen Size	8	x
	Splenomegaly	5	x
	Splenorenal	8	x
	Kidney – Normal	12	x
	Kidney – Hydro	8	x
	Kidney - CKD	5	
	Kidney - Mass	3	
	Kidney - Cyst	8	x
	Bladder Volume	8	x
	Prostate Normal	6	
	Prostate Enlarged	6	
	Uterus/Ovaries Normal	6	
	Uterus/Ovaries Abnormal	3	
	GB - Normal	20	x
	GB - Gallstones	8	x
	GB – Cholecystitis	6	
	GB - CBD dilated	6	
	GB - CBD normal	15	
	Hernia	5	
	SBO	5	
	Ileus	5	
**CARDIAC**			
	3-4 Views	50	x
	IVC Assessment	15	x
	Normal Function	15	x
	Hyperdynamic LV	8	x
	Severe Hypo LV	10	x
	Mild/Mod Hypo LV	18	x
	Segmental WMA	10	
	Pericardial Effusion	8	x
	Tamponade	5	
	RV Enlargement	10	x
	Left Pleural Effusion-Heart	5	x
	Right Pleural Effusion-Heart	3	
	Pleural and PCE	8	
	LV Wall Thickening	8	x
	Mitral Regurg	15	x
	Mitral Valve SAM	5	
	Mitral Stenosis/MAC	3	
	Tricuspid Regurg	15	x
	Aortic Regurg	15	x
	Aortic Stenosis	15	x
	Ao Root Dilation	10	
	Diastolic Normal	15	
	Diastolic Dysfunction	15	
**HEAD & NECK**			
	Sinus_NL	10	x
	Sinus_ABNL	3	x
	Thyroid – Normal	15	
	Thyroid – Abnormal	10	
	Ocular – Normal	10	
	Ocular – Abnormal	5	
	Optic Nerve Sheath	6	
**MUSCULOSKELETAL**			
	Knee Effusion	5	x
	Rib Fracture	4	
	Fracture (not rib)	5	
	Trochanteric Bursa	4	
	Baker's Cyst	4	
	Gout/Pseudo	4	
	Ganglion Cyst	3	
	Bursitis	5	
	Synovitis	5	
	Tendonitis/osis	5	
	Tear-Lig/Tendon	5	
	Tear-Muscle	5	
**PULMONARY**			
	Zones 1-4	15	x
	Zones 5-6	15	x
	Lung Sliding	10	x
	Pleural Effusion	10	x
	Interstitial Pattern	15	x
	Atelectasis	8	x
	Pneumonia/Consol	15	x
	Subpleural Consolidation	15	x
	Pneumothorax	5	x
**SOFT TISSUE**			
	Cellulitis	5	x
	Abscess	5	x
	Lymph N - Normal	8	x
	Lymph N - Abnormal	5	x
	Lymph N - Malignant	8	
	Cyst	8	
	Lipoma	8	
**VASCULAR**			
	IJ/CVP - Normal	8	
	IJ/CVP - Elevated	8	
	DVT Screen - Normal	25	
	DVT Screen - Abnormal	5	
	AAA Screen – Normal	25	
	AAA Screen - Abnormal	8	
	Carotid - Normal	15	
	Carotid - Plaque	8	
	Carotid - FlowTime	10	
